# Supplementary material for: HIVprotI: an integrated web based platform for prediction and design of HIV proteins inhibitors
Source: J Cheminform. 2018 Mar 9;10:12. doi: 10.1186/s13321-018-0266-y (PMC5845081; doi:10.1186/s13321-018-0266-y)
Supplement: Supplementary file 2 — Additional file 2. Source code of HIVProtI web server. [file 13321_2018_266_MOESM2_ESM.zip › HIVprotI_Source-code/hivprotI/weka-3-6-6/doc/weka/classifiers/bayes/net/package-summary.html]

weka.classifiers.bayes.net


---


|  |  |  |
| --- | --- | --- |
| **Overview** \| **Package** \| Class \| **Tree** \| **Deprecated** \| **Index** \| **Help** \| **Weka's home** | |  |
| **PREV PACKAGE**   **NEXT PACKAGE** | **FRAMES**    **NO FRAMES**     **All Classes** |  |


---

## Package weka.classifiers.bayes.net

| **Class Summary** | |
| --- | --- |
| **ADNode** | The ADNode class implements the ADTree datastructure which increases the speed with which sub-contingency tables can be constructed from a data set in an Instances object. |
| **BayesNetGenerator** | Bayes Network learning using various search algorithms and quality measures.  Base class for a Bayes Network classifier. |
| **BIFReader** | Builds a description of a Bayes Net classifier stored in XML BIF 0.3 format.    For more details on XML BIF see:    Fabio Cozman, Marek Druzdzel, Daniel Garcia (1998). |
| **EditableBayesNet** | Bayes Network learning using various search algorithms and quality measures.  Base class for a Bayes Network classifier. |
| **GUI** | GUI interface to Bayesian Networks. |
| **MarginCalculator** |  |
| **ParentSet** | Helper class for Bayes Network classifiers. |
| **VaryNode** | Part of ADTree implementation. |

---


|  |  |  |
| --- | --- | --- |
| **Overview** \| **Package** \| Class \| **Tree** \| **Deprecated** \| **Index** \| **Help** \| **Weka's home** | |  |
| **PREV PACKAGE**   **NEXT PACKAGE** | **FRAMES**    **NO FRAMES**     **All Classes** |  |


---
